# Vicenin-2 Hinders Pro-Inflammatory Response via Targeting the CaMKKβ-AMPK-SIRT1 Axis in Lipopolysaccharide-Stressed THP-1 Cells

**DOI:** 10.3390/ijms26052077

**Published:** 2025-02-27

**Authors:** Alessandro Maugeri, Caterina Russo, Giuseppe Tancredi Patanè, Martina Farina, Antonio Rapisarda, Mariorosario Masullo, Michele Navarra

**Affiliations:** 1 Department of Veterinary Sciences, University of Messina, I-98168 Messina, Italy; amaugeri@unime.it; 2 Department of Chemical, Biological, Pharmaceutical and Environmental Sciences, University of Messina, I-98166 Messina, Italy; carusso@unime.it (C.R.); giuseppe.patane@studenti.unime.it (G.T.P.); martina.farina@unime.it (M.F.); antonio.rapisarda@unime.it (A.R.); 3 Fondazione “Prof. Antonio Imbesi”, I-98123 Messina, Italy; 4 Department of Medical, Human Movement and Well-Being Sciences, University of Naples “Parthenope”, I-80133 Naples, Italy; mario.masullo@uniparthenope.it

**Keywords:** vicenin-2, flavonoid, inflammation, CaMKKβ, AMPK, SIRT1, docking studies

## Abstract

Plant secondary metabolites are known to be valuable agents to hamper inflammation owing to their multiple mechanisms of action. This study investigates the molecular mechanisms underlying the anti-inflammatory effects of vicenin-2 in lipopolysaccharide (LPS)-stressed THP-1 cells. After ascertaining the safety of vicenin-2 in our in vitro model, we assessed the anti-inflammatory potential of this flavonoid. Indeed, it counteracted the increase of tumor necrosis factor (TNF)-α, interleukin (IL)-1β, and IL-6 levels, as well as the overexpression of both inducible nitric oxide synthase (iNOS) and cyclooxygenase (COX)-2 caused by the exposure of THP-1 cells to LPS. Acknowledged the role of SIRT1 in the inflammatory process, we focused our attention on this enzyme. Our results showed that LPS dramatically decreased the expression of SIRT1, whereas vicenin-2 restored the levels of this enzyme to those of unexposed cells. These effects were also observed in terms of acetylated p53, a SIRT1 substrate. Notably, we observed that vicenin-2 did not act as a direct activator of SIRT1. Therefore, we investigated the potential involvement of AMP-activated protein kinase (AMPK), an upstream activator of SIRT1. Of note, by blocking AMPK by dorsomorphin, the protective effects of vicenin-2 on SIRT1 expression and activity were lost, suggesting the engagement of this kinase. Consequently, the blockage of AMPK caused a downstream loss of the anti-inflammatory effect of vicenin-2, which was no longer able to decrease both the activation of nuclear factor (NF)-κB and the production of cytokines induced by LPS. Finally, docking simulation suggested that vicenin-2 might act as an activator of Ca^2+^/calmodulin-dependent protein kinase kinase β (CaMKKβ), one of the regulators of AMPK. Overall, our results suggest that the anti-inflammatory effects of vicenin-2 may be due to the interaction with the CaMKKβ-AMPK-SIRT1 axis.

## 1. Introduction

Inflammation can be both a physiological and pathological response in the body. It serves as a defense mechanism against tissue or organ damage caused by physical, chemical, or biological stimuli. However, when the inflammatory event persists in an uncontrolled manner for a prolonged period, it turns into a pathogenic condition, thus becoming the cause of several chronic diseases. In this regard, it is well known that severe pathologies such as rheumatoid arthritis, atherosclerosis, diabetes, neurodegenerative diseases, infections, and cancer share an inflammatory component in their etiology [1]. Thereby, the role of inflammation as a key event of numerous pathogenetic processes has increased interest among researchers by prompting them to better elucidate the complexity of this phenomenon at the molecular level. Indeed, multiple signaling pathways jointly concur with the initiation of inflammation. Moreover, the inflammatory environment is closely influenced by an oxidative stress condition, which promotes or inevitably aggravates an inflammatory status [1].

Currently, the therapeutic strategies to counteract inflammation are limited to the selective inhibition of enzymes, receptors, or molecules [2]. However, a multi-target approach has been reviewed for synthetic and non-synthetic agents. This view has led, for example, to the observation of acetylsalicylic acid not only as a cyclooxygenase (COX) inhibitor, but also as being capable of interfering with mitogen-activated protein kinases (MAPKs), promoting the formation of lipoxins, acting on transcription factors like nuclear factor (NF)-κB, or activating AMP-activated protein kinase (AMPK) [3]. The latter is a key regulator of cellular energy, which maintains energy homeostasis via the coordination of several downstream pathways [4]. During inflammation, it is acknowledged that AMPK jointly cooperates with silent information regulator 1 (SIRT1), a NAD-dependent deacetylase to hamper pro-inflammatory signaling [5]. In particular, SIRT1 functions as a downstream protein of the AMPK cascade by inhibiting NF-κB, among other functions [6].

In the past, the potentiality of natural products was emphasized by “folk medicine”, whose mechanisms of action responsible for their effectiveness and their multi-target capability were not fully understood. Interestingly, more recently, compounds such as curcumin, epigallocatechin-3-gallate, resveratrol, and quercetin, revealed to be a potential attractive for developing anti-inflammatory multi-target drugs [7], thus encouraging new investigations from a mechanistic point of view. On the other hand, diet showed to exert an influence on different stages of inflammation through the regulation of multiple targets, such as enzymes or transcriptional factors involved in the release of pro-inflammatory mediators. In this context, polyphenolic compounds, such as flavonoids, which are widely present in fruits and vegetables, occupy a relevant place by jointly acting as antioxidant and anti-inflammatory agents [8,9,10]. In particular, dietary flavonoids like quercetin, genistein, apigenin, and kaempferol have been shown to modulate the expression and activation of pro-inflammatory cytokines. This occurred via the regulation of factors such as NF-κB, activator protein-1 (AP-1), as well as adhesion molecules (i.e., intercellular adhesion molecule-1 -ICAM-, vascular cell adhesion molecule-1 -VCAM-, and E-selectins). Moreover, dietary flavonoids have been also shown to hinder the synthesis of molecular messengers like nitric oxide and prostaglandins via the inhibition of inducible nitric oxide (NO) synthase, cyclooxygenase-2, and lipoxygenase [11,12].

Vicenin-2 is the 6,8-di-C-glucoside flavonoid of apigenin, isolated from different species, ranging from *Urtica circularis*, *Ocimum sanctum*, *Moringa oleifera*, and some *Citrus* varieties [13]. The extraction and identification of vicenin-2 as a phytochemical constituent of several medicinal plants led researchers to recognize relevant biological properties, including anti-inflammatory [14,15] and anticancer ones [16]. Regarding inflammation, the ability of vicenin-2 to modulate TET-2 in human macrophages [17], as well as to inhibit vascular permeability and expression of adhesive molecules, has been reported [18]. However, mechanisms leading to the anti-inflammatory effects of vicenin-2 have not been fully elucidated.

Therefore, the aim of our study was to investigate the upstream regulators influenced by vicenin-2 to exert its anti-inflammatory effect in THP-1 monocytes exposed to lipopolysaccharide (LPS), an in vitro model of acute inflammation, shedding light on the mechanisms involved.

## 2. Results

### 2.1. Vicenin-2 Does Not Induce Cytotoxic Effects in THP-1 Cells

**Figure 1:**
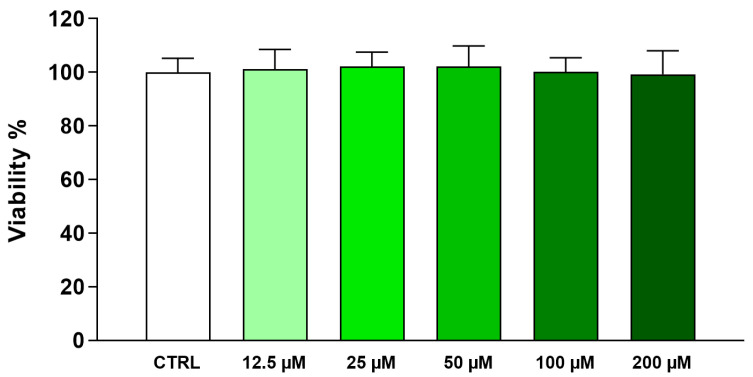
Effect of vicenin-2 on THP-1 cell viability. THP-1 cells were exposed to different concentrations of vicenin-2 (from 12.5 to 200 µM) for 24 h. Cell viability was assessed by the MTT test. Results are expressed as percentages of the values detected in untreated cultures containing DMSO as vehicle (CTRL). Data are expressed as means ± SEM of three independent experiments performed in eight replicates (*n* = 24).

The first step of this study was to assess the safety profile of vicenin-2 in THP-1 monocytes to select the concentrations to be further employed. The exposure of THP-1 cells to increasing concentrations of vicenin-2 (12.5–200 µM) for 24 h did not exert any sign of toxicity, as assessed by MTT test, a widely used method to evaluate the mitochondrial capacity of living cells (Figure 1). Therefore, the two highest concentrations were employed in the following experiments (i.e., 100 and 200 µM).

### 2.2. Vicenin-2 Reduces Gene Expression and Release of Pro-Inflammatory Cytokines in LPS-Stressed THP-1 Cells

**Figure 2:**
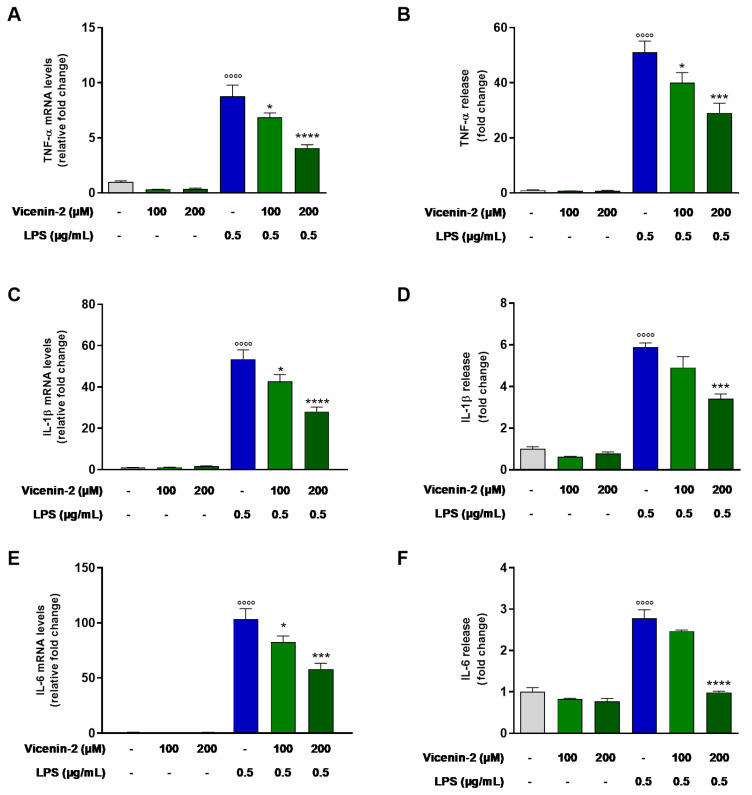
Effect of vicenin-2 on TNF-α, IL-1β, and IL-6 mRNA expression and release after LPS exposure of THP-1 cells. (**A**,**C**,**E**) Real-time PCR was employed to assess the levels of mRNA, and results are expressed as a relative fold change in exposed cells compared to those of untreated ones after normalization to β-actin. (**B**,**D**,**F**) Evaluation of secreted cytokines was carried out by ELISA in supernatants of THP-1 monocytes treated and untreated with vicenin-2 and LPS. Results are shown as fold change in cytokine release of exposed cells compared to that of untreated ones. (**A**–**F**) Data are expressed as the mean ± SEM of three experiments performed separately (*n* = 9). * *p* < 0.05, *** *p* < 0.001, and **** *p* < 0.0001 vs. LPS-exposed THP-1 cells (blue bar); °°°° *p* < 0.0001 vs. CTRL cells (light-gray bar).

The anti-inflammatory effect of vicenin-2 in LPS-exposed THP-1 cells was assessed in terms of mRNA expression and release of TNF-α, IL-1β, and IL-6 in the medium (Figure 2). The exposure of THP-1 cells to LPS alone for 3 h resulted in an increased transcription of *TNFA*, *IL1B*, and *IL6* (Figure 2A,C,E) compared to control cells. The pre-treatment with vicenin-2 decreased mRNA levels of TNF-α (*p* < 0.05 for 100 μM and *p* < 0.0001 for 200 μM; Figure 2A), IL-1β (*p* < 0.05 for 100 μM and *p* < 0.0001 for 200 μM; Figure 2C), and IL-6 (*p* < 0.05 for 100 μM and *p* < 0.001 for 200 μM; Figure 2E) in LPS-stressed THP-1 cells. These effects are reflected in terms of release of pro-inflammatory cytokines. Indeed, vicenin-2 was able to reduce the quantity of secreted TNF-α (*p* < 0.05 for 100 μM and *p* < 0.001 for 200 μM; Figure 2B), IL-1β (*p* < 0.001 for 200 μM; Figure 2D), and IL-6 (*p* < 0.0001 for 200 μM; Figure 2F) compared to LPS-exposed cells.

### 2.3. Vicenin-2 Hampers the mRNA Levels of iNOS and COX-2 in THP-1 Cells Exposed with LPS

**Figure 3:**
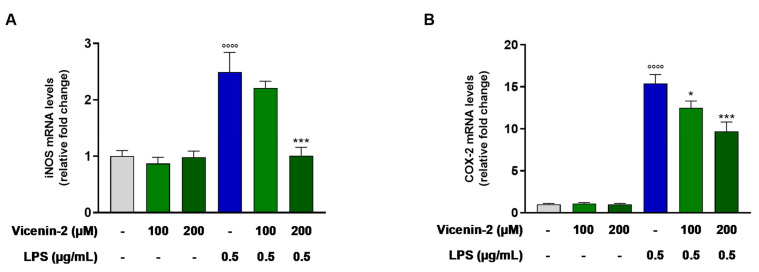
Levels of mRNA of iNOS (**A**) and COX-2 (**B**) in LPS-exposed THP-1 cells after vicenin-2 pre-treatment. Real-time PCR was employed to assess the levels of mRNA, and results are expressed as a relative fold change in exposed cells compared to those of untreated ones after normalization to β-actin. Data are expressed as the mean ± SEM of three experiments performed separately in triplicate (*n* = 9). * *p* < 0.05 and *** *p* < 0.001 vs. LPS-treated THP-1 cells (blue bar); °°°° *p* < 0.0001 vs. CTRL cells (light-gray bar).

Given the role of inducible nitric oxide synthase (iNOS) and cyclooxygenase-2 (COX-2), officially known as prostaglandin-endoperoxide synthase 2 (PTGS2), in the onset and sustainment of inflammation, we wondered whether the expression of these two enzymes was altered by the pre-treatment with vicenin-2. Indeed, it was able to significantly lower the mRNA levels of both iNOS (*p* < 0.05 for 100 μM and *p* < 0.01 for 200 μM; Figure 3A) and COX-2 (*p* < 0.01 for 100 μM and *p* < 0.001 for 200 μM; Figure 3B), which were dramatically increased by the exposure of LPS.

### 2.4. Vicenin-2 Restores the Gene Expression and Activity of SIRT1 After LPS Exposure in THP-1 Cells

**Figure 4:**
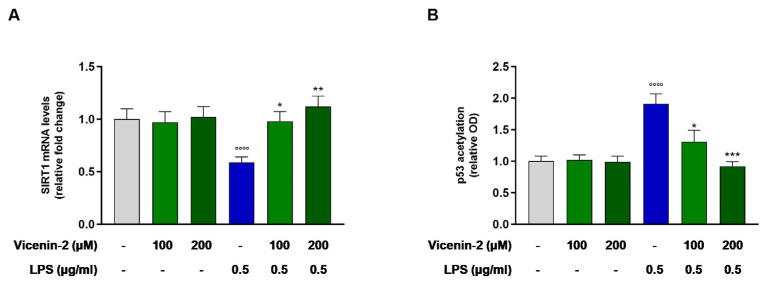
Effect of vicenin-2 on SIRT1 mRNA expression (**A**) and levels of acetylated p53 (**B**) after LPS exposure of THP-1 cells. Real-time PCR was employed to assess the levels of mRNA, and results are expressed as a relative fold change in exposed cells compared to those of untreated ones after normalization to β-actin (**A**). Relative protein levels of acetylated p53 were quantified by ELISA (**B**). Data are expressed as the mean ± SEM of three experiments performed separately in triplicate (*n* = 9). * *p* < 0.05, ** *p* < 0.01 and *** *p* < 0.001 vs. LPS-treated THP-1 cells (blue bar); °°°° *p* < 0.0001 vs. CTRL cells (light-gray bar).

Reports have disclosed the link between SIRT1 and inflammation, while the alterations of its expression and/or activity have been correlated to many inflammatory diseases [19]. Indeed, LPS was able to almost halve the mRNA levels of SIRT1 (*p* < 0.0001 vs. CTRL cells). Interestingly, vicenin-2 was able to revert this effect and brought back to levels of controls the mRNA of SIRT1 (*p* < 0.05 for 100 μM and *p* < 0.01 for 200 μM; Figure 4A). These effects were also reflected in terms of acetylated p53, one of the main substrates of the deacetylase activity of sirtuins (Figure 4B). Indeed, LPS increased the levels of acetyl-p53, due to a reduction in the expression and activity of SIRT1 (*p* < 0.001 vs. CTRL), whereas vicenin-2 was able to restore acetyl-p53 levels to that of controls (*p* < 0.05 for 100 μM and *p* < 0.001 for 200 μM; Figure 4B).

### 2.5. Vicenin-2 Does Not Directly Alter SIRT1 Deacetylase Activity

**Figure 5:**
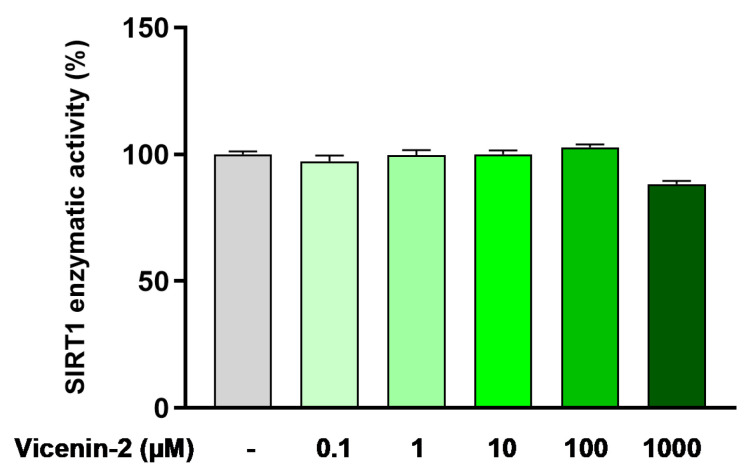
Cell-free SIRT1 enzymatic activity assessed after exposure to vicenin-2 (0.1–1000 µM). Control (CTRL, light-gray bar) consisted of the enzyme alone, while vicenin-2 (green bars) was tested at the shown concentrations. Data are expressed as the mean ± SEM of three experiments performed separately in triplicate (*n* = 9).

Based on the above results, we questioned whether the effect of vicenin-2 on SIRT1 was directly related to the activity of this enzyme. To this aim, we employed the recombinant isolated enzyme to assess whether vicenin-2 could directly act as an activator of SIRT1. Surprisingly, we witnessed that vicenin-2 did not alter the enzymatic activity at any of the concentrations employed (i.e., 0.1–1000 µM; Figure 5), thus suggesting that an upstream control on SIRT1 might be involved.

### 2.6. AMPK Is Involved in the Restoration of SIRT1 Gene Levels Elicited by Vicenin-2 in LPS-Treated THP-1 Cells

**Figure 6:**
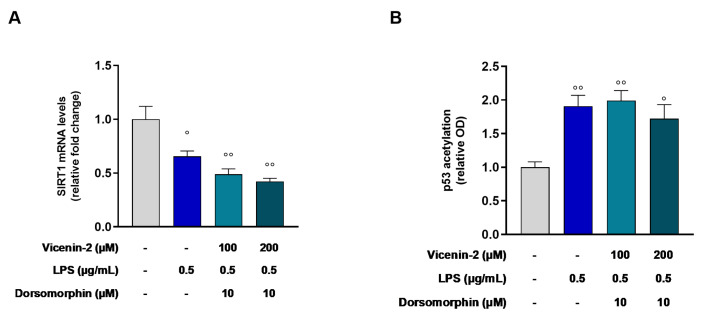
Blockage of the effect of vicenin-2 mediated by dorsomorphin on SIRT1 mRNA expression (**A**) and levels of acetylated p53 (**B**) after LPS exposure of THP-1 cells. (**A**) Real-time PCR was employed to assess the levels of mRNA, and results are expressed as a relative fold change in exposed cells compared to those of untreated ones after normalization to β-actin. (**B**) Relative protein levels of acetylated p53 were quantified by ELISA. Data are expressed as the mean ± SEM of three experiments performed separately in triplicate (*n* = 9). ° *p* < 0.05 and °° *p* < 0.01 vs. CTRL cells (light-gray bar).

The mutual correlation between AMPK and SIRT1 is acknowledged [20]. For this reason, we investigated AMPK involvement in the SIRT1 activation mediated by vicenin-2 in this in vitro model. To this aim, we employed dorsomorphin, an AMPK inhibitor, together with vicenin-2, before the LPS stimulus. As shown in Figure 6, the blockage of AMPK activity by dorsomorphin caused a loss of the effects induced by vicenin-2 on SIRT1 enzyme, for both 100 and 200 µM (*p* < 0.01 vs. CTRL cells). This occurred not only in terms of mRNA levels (Figure 6A) but also in terms of acetylated p53, as quantified by ELISA assay (*p* < 0.01 and *p* < 0.05 for 100 and 200 µM vs. CTRL cells, respectively; Figure 6B).

### 2.7. The Blockage of AMPK Hampers the Anti-Inflammatory Potential of Vicenin-2

**Figure 7:**
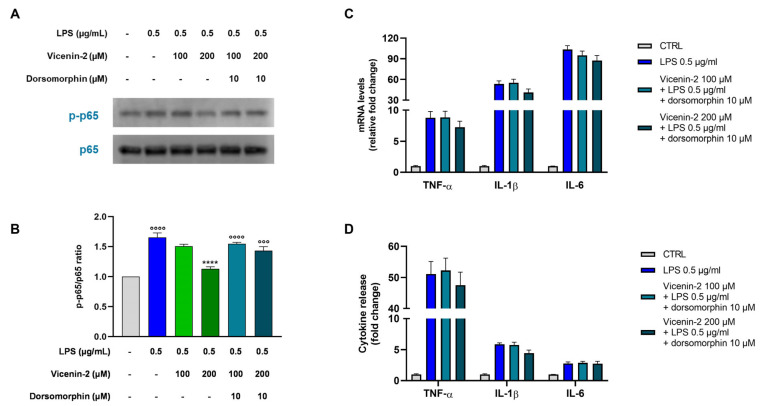
Role of dorsomorphin in counteracting the anti-inflammatory effect of vicenin-2 in LPS-treated THP-1 cells. (**A**) Representative immunoblots of the p65 phosphorylation status of three separate experiments are shown. (**B**) Densitometric analysis of bands from three independent blots is presented. The results are expressed as the ratio of phosphorylated form compared to the total protein (p-p65/p65), and compared to the untreated cells (CTRL, light-gray bar), which are arbitrarily assigned as 1. Results are expressed as fold change compared to the untreated cells. (**C**) Real-time PCR was employed to assess the levels of mRNA of cytokines, and results are expressed as a relative fold change in exposed cells compared to those of untreated ones after normalization to β-actin. (**D**) Evaluation of secreted cytokines was carried out by ELISA in supernatants of THP-1 monocytes treated and untreated with vicenin-2 and LPS. Results are shown as fold change in cytokine release of exposed cells compared to that of untreated ones. Data are expressed as the mean ± SEM of three experiments performed separately (for Western blotting, *n* = 3, while for RT-PCR and cytokine assays, *n* = 9). **** *p* < 0.0001 vs. LPS-stressed THP-1 cells (blue bar); °°° *p* < 0.001 and °°°° *p* < 0.0001 vs. CTRL cells (light-gray bar).

Since AMPK plays a key role in inflammation, we next examined whether blocking AMPK affects the downstream inflammatory response. For this reason, we focused our attention on the NF-κB transcription factor, a pivotal element of the inflammatory response [21]. Therefore, we evaluated the phosphorylation levels of the subunit RelA (p65) of the NF-κB family, which is commonly regulated by histone deacetylases such as SIRT1 [22]. As expected, the exposure of THP-1 cells to LPS dramatically enhanced the levels of p-p65 compared to untreated cells (*p* < 0.0001 vs. CTRL, Figure 7A,B, blue bar). The pre-treatment with vicenin-2 at a concentration of 100 µM was not able to significantly counteract the LPS-induced effect in THP-1 cells (light green bar), while the 200 µM (dark green bar) significantly reduced the p-p65 protein expression (*p* < 0.0001 vs. LPS-treated cells) to almost control levels. Interestingly, the presence of the AMPK inhibitor totally reverted the protective effect induced by vicenin-2 by restoring p-p65 levels to those of the cells exposed to the LPS alone (*p* < 0.0001 vs. CTRL; cyan bars), thus suggesting an action of vicenin-2 on AMPK.

Moreover, knowing that NF-κB is crucial for the expression of pro-inflammatory mediators [21], we assessed the expression and release of TNF-α, IL-1β, and IL-6 after the blockage of AMPK (Figure 7C,D). Notably, dorsomorphin abolished the protective effect of vicenin-2 against NF-κB activation induced by LPS. This resulted in the maintenance of elevated cytokine levels in cells pre-treated also with vicenin-2, similar to those detected in cells exposed to LPS alone, confirming the role of AMPK in the observed effects.

### 2.8. CaMKKβ Is a Potential Target of Vicenin-2 to Activate AMPK

**Figure 8:**
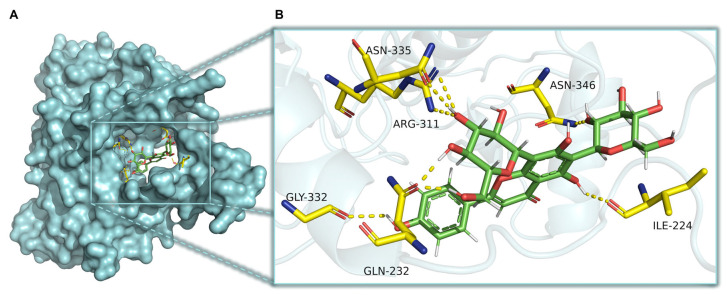
Interaction of vicenin-2 in the active site of CaMKKβ determined in silico. (**A**) Best docked pose of vicenin-2 (in green; PDB code 2ZV2) within the CaMKKβ protein (in cyan surface; PDB code 2ZV2). (**B**) Interactions of vicenin-2 at selectivity pocket of CaMKKβ. Crucial residues are shown in yellow sticks, while hydrogen bonds are shown as dashed lines.

The Ca^2+^/calmodulin-dependent protein kinase kinase β (CaMKKβ) is a crucial signaling protein that controls inflammation by increasing intracellular calcium. Indeed, it has been disclosed that the CaMKKβ/AMPK/SIRT1 axis is linked to the regulation of the release of cytokines, after the occurrence of an inflammatory stimulus [23]. For this reason, we investigated, via docking simulation, the potential binding of vicenin-2 in the allosteric site present in CaMKKβ. Interestingly, the best docked pose of the flavonoid showed a free binding energy of −7.866 kcal/mol. Moreover, the best docked pose was able to create a dense network of hydrogen bonds. Indeed, the sugar moieties interacted with Asn335, Arg311, Gln232, and Asn346, whereas the aglycone moiety interacted with Ile224 and Gly332 (Figure 8).

## 3. Discussion

The plant kingdom has always represented fertile soil for novel chemical entities endowed with interesting biological properties, such as anti-inflammatory ones [24]. Noteworthy is the story of salicylates, which are among the first natural compounds employed in traditional medicine for their anti-inflammatory potential [3]. Although the medicinal properties of willow bark extracts have been recognized for about two millennia, the use of sodium salicylate in the treatment of rheumatic illness was widespread only 120 years ago. It took a further seventy-two years for Vane and collaborators to prove that aspirin-like medications work by inhibiting prostaglandin formation [3]. This acquired knowledge allowed the progressive discovery of a number of other uses of salicylates as an irreversible inhibitor of the COX enzymes. Then, it allowed for the creation of chances for novel therapies for cardiovascular and neurodegenerative diseases, as well as cancer [25]. Therefore, it is crucial to assess the mechanisms underlying the effects of natural compounds in order to exploit their full potential. This is even more challenging for compounds like flavonoids, which are a class of numerous entities with uncountable biological targets [12].

On this line, this study was aimed at the assessment of the pathways involved in the anti-inflammatory potential of vicenin-2. This flavone-C-glycoside has been shown to possess interesting anti-inflammatory effects both in vitro [15,17] and in vivo [14]. Interestingly, these studies agreed that vicenin-2 was able to down-regulate the activation of NF-κB, which hence lowered cytokines release, reactive oxygen species, or inflammasome activation. The missing point was the upstream element influencing NF-κB, the keystone of inflammation. In this study, we employed undifferentiated THP-1 monocytes, which are a sensitive in vitro model that resembles the first stages of inflammation [26].

In our model, we challenged THP-1 monocytes with LPS, which prompted a significant production of pro-inflammatory cytokines, including IL-1β, IL-6, and TNF-α, generally regarded as key indicators of the inflammatory response. They also play a role in activating the NF-κB signaling pathway, which in turn stimulates the transcription of a number of genes that are known to exacerbate the inflammatory response [27]. Interestingly, vicenin-2 was able to revert this effect, lowering the expression and release of TNF-α, IL-6, and IL-1β, in line with previous reports [14,15,17]. In this context, vitexin, a flavone C-glycoside, demonstrated anti-inflammatory effects via the reduction of both TNF-α and IL-6 levels in rat chondrocytes and in chondrocytes from osteoarthritis patients [28,29].

The overexpression of NO synthase, specifically the inducible form (iNOS), is an important indicator of LPS-induced stress, which leads to the excessive production of nitric oxide and, ultimately, inflammation [30]. Similarly, COX-2 upholds the release of the pro-inflammatory factors, aiding the inflammatory response [31]. In our study, we observed a rise in the expression of both *NOS2* and *PTGS2* in THP-1 cells stressed with LPS. In contrast, vicenin-2 significantly reduced the overexpression of these genes induced by LPS, thus exhibiting anti-inflammatory effects. Also, in this case, our results are in line with what was previously reported regarding the anti-inflammatory potential of vicenin-2 [14,15,17]. Similarly, orientin, another flavone C-glycoside, was able to lower mRNA levels of both COX-2 and iNOS, as well as other pro-inflammatory markers, in LPS-challenged C57BL/6 mice as a model of acute lung injury [32].

Recent studies have suggested that the regulation of both innate immunity and energy metabolism is linked together via an antagonistic crosstalk between NF-κB and SIRT1. This is because the former can stimulate glycolysis during acute inflammation, whereas the latter inhibits NF-κB signaling to promote the resolution of inflammation [33]. Based on this premise, we focused on this step prior to NF-κB in the inflammatory cascade to understand whether vicenin-2 could act on SIRT1. Interestingly, we found that this flavonoid could effectively restore the expression of this sirtuin back to levels of unstressed cells and, consequently, to restore the level of acetylated p53, meaning a renewal of SIRT1 activity. Many flavonoids have been suggested to possess SIRT1-promoting capabilities, helpful to counteract inflammation in several degenerative diseases [34,35,36], as well as flavonoid-C-glycosides [37].

Based on these results, we assessed the effect of vicenin-2 on the recombinant isolated enzyme to comprehend whether we could witness a direct effect on SIRT1 deacetylase activity. Surprisingly, vicenin-2 had no effect on the enzymatic activity of SIRT1 at any concentration we studied. Therefore, we focused on another element tightly linked to SIRT1, namely AMPK. This kinase regulates SIRT1, and together, AMPK and SIRT1 play pivotal roles in both energy balance and inflammatory response [38,39]. AMPK activation caused a negative regulation of inflammation via the blockage of NF-κB, the expression of inflammatory genes, and the whole inflammatory damage [40]. To study its involvement, we employed the AMPK inhibitor dorsomorphin, also known as compound C. By blocking the activity of AMPK, we witnessed a complete loss of the effect of vicenin-2 on both SIRT1 expression and activity, suggesting the involvement of this kinase. In the literature, there are reports of flavonoid-C-glycosides able to interact with this pathway, like orientin (luteolin-8-C-glucoside). This compound was shown to possess antioxidant capacity and support mitochondrial functionality in both in vitro and in vivo models via activation of the AMPK/SIRT1 pathway [41,42]. Moreover, we witnessed a loss of the protection against the activation of NF-κB, which is the key regulator of the whole inflammatory process [21]. Indeed, dorsomorphin hindered the effects of vicenin-2 in the blockage of NF-κB phosphorylation, which led to a loss of the capability of vicenin-2 to also lower cytokine levels.

According to these findings, we wondered whether vicenin-2 could act as an AMPK activator. Previous reports have supported docking simulation as a further consistent strategy to assess AMPK activators [43]. By employing this technique, we found out that vicenin-2 could not bind to the activator site of AMPK, as suggested by others for natural chalcone derivatives [44]. Nevertheless, a study from Liu and collaborators, who showed that isovitexin, a flavonoid-C-glycoside, exerted anti-inflammatory effects in LPS-exposed microglial cells via the CaMKKβ/AMPK axis [45], prompted us to investigate this kinase. Interestingly, docking simulations have shown that vicenin-2 could effectively bind to the activator site of CaMKKβ, thus suggesting that this could be a potential target of the anti-inflammatory effect of vicenin-2.

## 4. Materials and Methods

### 4.1. Cell Culture

Human leukemia monocytic THP-1 was originally obtained from ATCC (Rockville, MD, USA), cultured at 37 °C in a 5% CO_2_-air humified atmosphere in RPMI 1640 supplemented with 10% (*v*/*v*) heat-inactivated fetal bovine serum (FBS), L-glutamine (2 mM), HEPES (10 mM), sodium pyruvate (1 mM), glucose (2.5 g/L), 2-mercaptoethanol (0.05 mM), penicillin (100 IU/mL), and streptomycin (100 µg/mL). Each reagent for cell culture was from EuroClone (Milan, Italy). This cell line was chosen based on previous reports suggesting its robustness for investigating inflammatory processes in vitro [46]. Vicenin-2 was obtained from MedChemExpress, (DBA Italia, Milan, Italy) and resuspended in DMSO at a final concentration of 100 mM. Stock solutions were kept at −20 °C and defrosted prior use. Dorsomorphin was from Selleckchem (Houston, TX, USA). We employed this compound, being an acknowledged potent, reversible, and selective AMPK inhibitor [47].

### 4.2. Safety Profile of Vicenin-2

To assess the safety of vicenin-2, we performed a 3-(4,5-dimethylthiazole-2-yl)-2,5-diphenyltetrazolium bromide (MTT) test [48]. Briefly, THP-1 cells were seeded in 96-well plates at a density of 5 × 10^4^ cells/well. The day after, cells were incubated with fresh medium containing 0.2% (*v*/*v*) DMSO as vehicle (untreated cells) or with medium supplemented with increasing concentrations of vicenin-2 (1–200 µM) in which DMSO was set to 0.2% (*v*/*v*) final concentration. After further 24 h, plates were centrifuged, and supernatants were removed and incubated with fresh medium (phenol red-free) containing 0.5 mg/mL of MTT (Sigma-Aldrich, Milan, Italy) at 37 °C for 4 h. Afterward, the precipitated formazan crystals were dissolved in 100 µL of a 0.1 N HCl/isopropanol lysis solution. The absorbance of each well was measured at a wavelength of 570 nm, using a microplate reader (Bio-Rad Laboratories, Milan, Italy). Results were expressed as cell viability percentage compared to untreated cells, which were arbitrarily set as 100%. All experiments were performed in eight replicates and repeated three times.

### 4.3. Evaluation of Cytokine Secretion

THP-1 cells were seeded in Petri dishes (5 × 10^5^ cells/mL) and treated with vicenin-2 at 100 and 200 µM for 30 min, with or without 10 µM of dorsomorphin, before being exposed to lipopolysaccharide (500 ng/mL; Sigma-Aldrich) for a further 3 h. The secretion of IL-6, IL-1β, and TNF-α was assessed via an enzyme-linked immunosorbent assay on supernatants of treated-THP-1 cells. Prior to detection, the supernatants recovered were concentrated ten-fold by freeze-drying. All freeze-dried samples were reconstituted by the addition of bi-distilled water. Following manufacturer’s guidelines, 50 μL of standards or samples were incubated in 96-well plates for 3 h under constant shaking at room temperature. After washing five times with 400 μL of wash buffer, 100 μL of the provided substrate solution was added to each well, and plates were incubated in the dark for 10 min. The enzyme reaction was then stopped by adding 100 μL of stop solution to each well. Absorbance was determined at 450 nm, using a microplate reader (Bio-Rad Laboratories). The experiments were performed in triplicate, and results are expressed as fold change compared to untreated cells [49].

### 4.4. Real-Time PCR Analysis

For the evaluation of gene expression, THP-1 cells were treated as described above. Cells were harvested by centrifugation, and total RNA was isolated using TRIzol (Invitrogen, Carlsbad, CA, USA), as shown previously [49]. An equal amount of total RNA (2 µg) for each sample was reverse-transcribed into cDNA, employing the High-Capacity cDNA Archive Kit (Applied Biosystems, Life Technologies, Foster City, CA, USA). Afterward, quantitative PCR reaction (qPCR) was carried out via a 7500 qPCR System (Applied Biosystems), in a total volume of 20 µL, including 1x SYBR^®^ Select Master Mix (Applied Biosystems), 0.2 µM of specific primers, and 25 ng of RNA converted into cDNA. Data were analyzed using the 2^−∆∆CT^ relative quantification method versus β-actin (ACTB), used as endogenous control. The values are presented as n-fold change with respect to untreated cells. The primer sequences used for real-time PCR are listed in Table 1.

### 4.5. Quantification of Acetylated p53 in THP-1-Treated Cells

In order to assess the levels of acetylated p53 in LPS-exposed THP-1 cells treated with vicenin-2, we employed a commercial enzyme-linked immunosorbent assay (ELISA) kit (E4531; Biovision, Milpitas, CA, USA) [50]. Briefly, cell lysates were quantified using Bio-Rad DC Protein Assay (Bio-Rad Laboratory, Hercules, CA, USA), with the aim of testing the same amount of protein for each condition. Afterwards, 100 µL of biotin-conjugated primary antibody was added in provided strips and incubated at 37 °C for 1 h. After wash, 100 µL of streptavidin HRP-conjugated was added and incubated for further 30 min at 37 °C. Latterly, 90 μL of TMB substrate was added to each well, and the plate was incubated at 37 °C in the dark for 30 min. Color formation was stopped by adding stop solution, and absorbance was recorded with a microplate spectrophotometer at 450 nm (iMark™ microplate reader, Bio-Rad Laboratories). Results are expressed as the ratio between values detected in treated and untreated cells.

### 4.6. SIRT1 Histone Deacetylase Activity Assay

SIRT1 activity assay was performed using a fluorometric kit purchased from Enzo Life Science (Farmingdale, NY, USA), according to the manufacturer’s protocol [51]. Increasing concentrations of vicenin-2 (from 0.1 to 1000 µM) were tested employing the recombinant enzyme provided by the kit. Briefly, the Substrate Solution containing 2 mM nicotinamide adenine dinucleotide (NAD) and 125 μM peptide was added to the reaction mixture and incubated for 45 min at 37 °C. Afterward, a Stop/Developing Solution consisting of developer plus nicotinamide was added to each well for 30 min. Finally, fluorescence was acquired using a FLUOstar Omega Plate Reader (BMG LABtech, Ortenberg, Germany) at 350–360 nm excitation wavelength and 450–465 nm emission wavelength.

### 4.7. Assessment of NF-κB Phosphorylation

For the evaluation of NF-κB phosphorylation, THP-1 cells were grown in 100 mm Petri dishes (1 × 10^6^ cells/dish) and treated as explained above. Afterward, cells were harvested, washed with PBS, and lysed using RIPA buffer (Sigma-Aldrich) supplemented with 1% cocktail protease and phosphatase inhibitors (Sigma-Aldrich). Then, lysed cells were centrifuged at 12,000× *g* for 15 min at 4 °C, and the supernatant was recovered. The protein concentration of supernatant was determined using Bio-Rad DC Protein Assay (Bio-Rad Laboratory), using BSA as protein standard. Equal amounts of proteins (30 μg/lane) were separated by 10% sodium dodecyl sulphate–polyacrylamide gel electrophoresis (SDS-PAGE) and electro-transferred on a polyvinylidene fluoride (PVDF) (GVS Life Sciences, ME, USA), where non-specific binding sites were blocked with 5% (*w*/*v*) non-fat dry milk for 1 h at room temperature. Then, membranes were incubated at 4 °C overnight with primary antibodies for total or phosphorylated p65 (1:1000 in milk; Cell Signaling Technology, Danvers, MA, USA). Membranes were then washed thrice in Tris-Buffered Saline containing 0.15% of Tween 20 (TBS-T) and incubated with horseradish peroxidase-conjugated goat anti-rabbit IgG secondary antibody (1:2500, Sigma-Aldrich) for 2 h at room temperature. Chemiluminescence of protein bands was achieved using Luminata Forte Western HRP Substrate (Millipore, MA, USA) and visualized by a chemiluminescent detection system C-Digit Blot Scanner (Li-COR Bioscience, Lincoln, NE, USA). Protein bands were quantified using Image Studio 6.0 software (Li-COR Bioscience) as ratio between phosphorylated and total p65 [52].

### 4.8. Statistical Analysis

Data from the experiments performed in triplicate were expressed as mean ± standard error of the means (SEM) and statistically assessed for differences using one-way analysis of variance (ANOVA), followed by the Dunnett’s multiple comparison test (GraphPad Prism 8.4.2 Software, San Diego, CA, USA). The *p*-values less than or equal to 0.05 were considered significant.

### 4.9. Docking Studies

The ligand vicenin-2 was designed using the Maestro 14.0 software (Schrödinger LLC; New York, NY, USA) and minimized to produce a low-energy 3D structure. The docking studies were performed by GOLD software (Hermes 2024.1.0, Cambridge Crystallographic Data Centre; Cambridge, UK). The 3D structure of human CaMKKβ was retrieved from the Protein Data Bank consisting of the co-crystal structure of CaMKKβ kinase with the synthetic inhibitor STO-609 inhibitor (2ZV2), while the missing residues of P-loop and αC-helix were constructed by Phyre2 homology modeling, as described in [44,53]

Protein structure was freed by crystallized ligands and water molecules, while hydrogens were added by Maestro 14.0 software (Schrödinger LLC). A pH range of 7.2–7.4 was used to mimic physiological condition. Docking simulations were performed by choosing the coordinates of the allosteric site in CaMKKβ to contain the residues within 10 Å within this position. The ChemPLP was employed as fitness functions, and the ligand was submitted to 100 genetic algorithm runs. Results differing by less than 0.75 Å in ligand-all atom RMSD were clustered together. The SwissDock program was employed to retrieve another algorithm of docking simulation [54,55]. The interactions of the best ranked conformation of vicenin-2 with the surrounding residues, along with the images, were extrapolated by PyMOL 2.6.0 software (Schrödinger LLC) [56]. 

## 5. Conclusions

**Figure 9:**
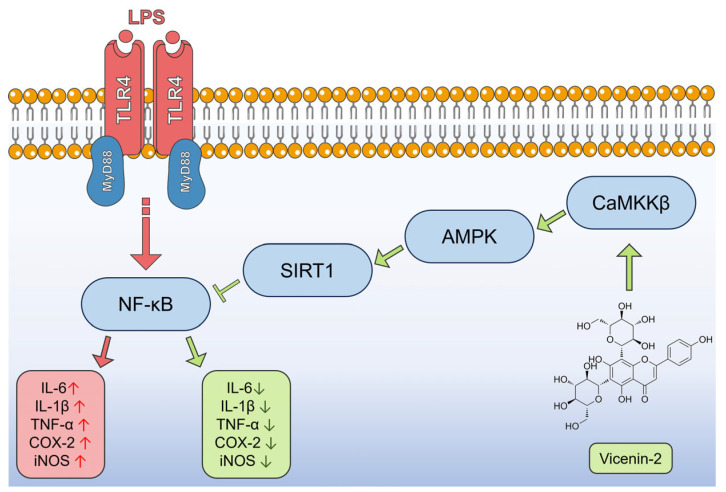
Putative mechanism underlying the anti-inflammatory effect of vicenin-2 in LPS-stressed THP-1 cells. Red arrows represent the cascade activated by LPS, while green arrows represent the potential path followed by vicenin-2 to hamper inflammation.

In this study, we proved that the anti-inflammatory effect of vicenin-2 in our in vitro model of acute inflammation is due to an interplay of factors (Figure 9). Indeed, we showed that vicenin-2 is able to restore the levels and activity of SIRT1 that is hampered by LPS exposure. Moreover, we indicated that the blockage of AMPK caused the loss of the protective effects of vicenin-2 on SIRT1 enzyme, as well as to the downstream inflammatory cascade. In silico analyses pointed out the potential role of vicenin-2 as an activator of CaMKKβ. Overall, our results suggest the involvement of the CaMKKβ/AMPK/SIRT1 axis in the anti-inflammatory potentiality of vicenin-2 and support the need of further investigations in more complex models to shed light on the other potential elements contributing to the observed effects.

## Figures and Tables

**Table 1:** Oligonucleotide primer sequences employed for real-time PCR.

Gene	Name	NCBI Reference Sequence	Primer Sequence
*ACTB*	Actin beta	NM_001101.5	Forward: 5-TTGTTACAGGAAGTCCCTTGCC-3′ Reverse: 5′-ATGCTATCACCTCCCCTGTGTG-3′
*IL1B*	Interleukin 1 beta	NM_000576.3	Forward: 5′-AGCCATGGCAGAAGTACCTG-3′ Reverse: 5′-TGAAGCCCTTGCTGTAGTGG-3′
*IL6*	Interleukin 6	NM_000600.5	Forward: 5′-CCACCGGGAACGAAAGAGAA-3′ Reverse: 5′-GAGAAGGCAACTGGACCGAA-3′
*NOS2*	Nitric oxide synthase 2	NM_000625.4	Forward: 5′-GGATGACAACCGATACCA-3′ Reverse: 5′-GAAGGCAATGGACTCAGA-3′
*PTGS2*	Prostaglandin-endoperoxide synthase 2	NM_000963.4	Forward: 5′-GCCTGGTCTGATGATGTA-3′ Reverse: 5′-TCTGGAACAACTGCTCAT-3′
*SIRT1*	Sirtuin 1	NM_012238.5	Forward: 5’-AACTACTTCGCAACTATACC-3′ Reverse: 5′- ACCATGACACTGAATTATCC-3′
*TNFA*	Tumor necrosis factor alpha	NM_000594.4	Forward: 5′-CACAGTGAAGTGCTGGCAAC-3′ Reverse: 5′-ACATTGGGTCCCCCAGGATA-3′

## Data Availability

The original contributions presented in this study are included in the article. Further inquiries can be directed toward the corresponding author.
